# Construction of a circRNA-Related Prognostic Risk Score Model for Predicting the Immune Landscape of Lung Adenocarcinoma

**DOI:** 10.3389/fgene.2021.668311

**Published:** 2021-08-09

**Authors:** Huawei Li, Jun Wang, Linyou Zhang

**Affiliations:** Department of Thoracic Surgery, The Second Affiliated Hospital of Harbin Medical University, Harbin, China

**Keywords:** lung adenocarcinoma, circRNA, targeted therapy, chemotherapy, immune cells

## Abstract

The purpose of this study was to construct a circular RNA (circRNA)-related competing endogenous RNA (ceRNA) regulatory network and risk score model for lung adenocarcinoma (LUAD). The relationship of the risk score to immune landscape and sensitivity to chemotherapy and targeted therapy of LUAD was assessed. We downloaded mRNA and miRNA expression data, along with clinical information, from The Cancer Genome Atlas (TCGA) program, and circRNA expression data from the Gene Expression Omnibus (GEO) database and identified differently expressed circRNA (DEcircRNA), miRNA (DEmiRNA), and mRNA (DEmRNA) using R software. We then constructed the circRNA-related network using bioinformatics method. The risk score model was established by LASSO Cox regression analysis based on 10 hub genes. In addition, the risk score model was an independent predictor for overall survival (OS) in both the TCGA and CPTAC datasets. Patients in the high-risk group had shorter OS and disease-free survival (DFS) than those in the low-risk group and were more sensitive to chemotherapy and targeted therapy. The types of tumor-infiltrating immune cells were different in the high- and low-risk groups. Our data revealed that the circRNA-related risk score model is closely associated with the level of immune cell infiltration in the tumor and the effects of adjuvant treatment. This network may be useful in designing personalized treatments for LUAD patients.

## Introduction

Worldwide, lung cancer is a leading cause of cancer-related deaths, and approximately half of cancers are lung cancers (Imielinski et al., 2012; Bray et al., 2018). Since most lung cancer patients are diagnosed at an advanced stage, the 5-year survival rate is only about 18%, even if diagnosis and treatment were improved (Siegel et al., 2018). Therefore, exploring the molecular mechanism of lung adenocarcinoma (LUAD) and establishing an effective prognostic model for this cancer are critical in the formulation of effective individualized treatment regimens.

Circular RNA (circRNA) derived from gene intron or exon region are a special type of non-coding RNA. They have a closed circular structure and no poly-A tail. Therefore, compared with linear RNA, circRNAs have a more stable structure and are not easily hydrolyzed by exonuclease or RNase (Wang et al., 2016). The competing endogenous RNA (ceRNA) hypothesis holds that circRNAs can compete with mRNA, the downstream targets of microRNAs (miRNAs), to bind miRNA response elements and, in turn, affect mRNA expression levels, thus forming a complex posttranscriptional regulatory mechanism (Salmena et al., 2011). To explore the potential function and mechanism of circRNA in LUAD, we established the circRNA-related ceRNA regulatory network. Based on the identification of downstream mRNAs, we then generated a prognostic risk score model.

Previous studies demonstrated that circRNA participates in the regulation of immune cell infiltration in the tumors through the ceRNA mechanism (Song et al., 2020). Therefore, we also explored the relationship between the risk score and the level of immune cell infiltration and assessed the relationship between the risk score and the immunosuppressive molecules.

Currently, adjuvant therapy planning after tumor resection is mainly designed according to TNM stage (Amin et al., 2017). Due to the tumor heterogeneity, adjuvant treatment plans based only on TNM stage have certain limitations. Therefore, we predicted the sensitivity of LUAD patients to chemotherapy and targeted drugs according to the risk score.

In this study, we first constructed a circRNA-related ceRNA network through bioinformatics analysis, then constructed a prognostic risk score model. Finally, we explored the relationship between risk score and the level of infiltrated immune cells in LUAD, genes related to immune checkpoint inhibitors (ICIs), and sensitivity of chemotherapy and targeted therapy.

## Materials and Methods

### Data Collection and Preprocessing

Two circRNA expression datasets GSE101684 and GSE112214 were obtained from GEO database.^1^ The normalizeBetweenArrays function in the ‘‘Limma’’^2^ package in R software was used to normalize the expression data of circRNA, and the batch effect was corrected by using ComBat function in “sva” package in R software after merging the two datasets (Leek et al., 2012). Linear fitting was performed on the data by using lmFit function. Finally, the mean expression value of 3,468 circRNAs in LUAD tissues and paracancerous tissues were analyzed by using empirical eBayes in the “Limma” package to determine the differentially expressed circRNAs (DEcircRNAs) based on a screening criteria of false-discovery rate (FDR) <0.05 and | log2 fold change(FC)| >1. However, we did not consider the paired nature of the circRNA data when we analyzed the differentially expressed genes. The ‘‘pheatmap’’^3^ package was used to visualize the DEcircRNAs, whose expression value had been normalized.

Clinical information of 522 LUAD patients and the expression data of miRNA (513 tumor and 46 paracancerous samples) and mRNA(513 tumor and 59 paracancerous samples) were acquired from The Cancer Genome Atlas (TCGA).^4^ Fifty LUAD patients were excluded from this research, because of unknown age (10 patients), no or less than 30 days of survival time (23 patients), no tumor stage (eight patients), and no mRNA expression data (nine patients). Finally, 472 LUAD patients with complete clinical information were included in our study. Clinical Proteomic Tumor Analysis Consortium (CPTAC)^5^ datasets containing clinical information and RNA sequencing data of 102 LUAD patients were obtained for external validation of the risk score model. Low-expressing mRNAs with an average read counts of <5 and low expressing miRNAs with an average read counts of <1 were filtered out. The 17,143 mRNAs and 817 miRNAs meeting the above requirements were included in this analysis. For the raw read counts of mRNA and miRNA, the calcNormFactors function in the “edgeR” package (Robinson et al., 2010) in R software was used to calculate the normalization factors in each sample to normalize the gene expression data. The exactTest function was used to identify the differentially expressed genes based on the screening criteria of FDR <0.05 and |log2 fold change (FC)| >1. For miRNA and mRNA correlation analyses, we transform the read count matrix of miRNA and mRNA into a matrix of transcripts per million (TPM) values.

### Constructing the ceRNA Network

The target DEmRNAs of the DEmiRNAs were predicted using the miRTarBase and TargetScan databases (Hsu et al., 2011; Agarwal et al., 2015). To improve the reliability, the coexpression relationship of the DEmiRNA and DEmRNA from the DEmiRNA/DEmRNA pairs predicted by two database were further analyzed by Spearman’s correlation analysis screened according to a criteria of the Spearman’s correlation coefficient (ρ) < –0.2, FDR <0.05, and the standard deviation (sd) >0.5. We named these gene pairs NC-DEmiRNAs/DEmRNAs pairs. The target miRNAs of DEcircRNA were predicted using the circBank database,^6^ then we took the intersection of these targeted miRNA and DEmiRNAs from the NC-DEmiRNAs/DEmRNAs pairs. The expression patterns between circRNA and miRNA of a circRNA/miRNA pairs must be opposite, that is, if a circRNA expression is upregulated, the corresponding miRNA must be downregulated, and vice versa. According to the above result, we utilized Cytoscape (version 3.7.2) to construct a circRNA/miRNA/mRNA network.

### Functional Enrichment Analysis

We performed Gene Ontology (GO) function and Kyoto Encyclopedia of Genes and Genomes (KEGG) pathway analyses on these 122 DEmRNAs in this network to evaluate their enrichment for biological processes (BP), molecular function (MF), and cellular component (CC) and to annotate their signal pathways. The ‘‘clusterProfiler’’^7^ package was used to perform GO and KEGG analysis based on the screening criteria of adjusted p (*q*-value) <0.05.

### Protein-Protein Interaction Network

Using the Search Tool for the Retrieval of Interacting Genes (STRING) database,^8^ the interaction of these DEmRNAs from the circRNA/miRNA/mRNA networks were explored. Interactions among proteins with a comprehensive score >0.7 were thought to be statistically significant. Then, we established a protein-protein interaction (PPI) network for these DEmRNAs using STRING and visualized it with Cytoscape. The CytoHubba application was used to extract hub genes from the PPI network according to the degree method.

### Survival Prediction Model of Hub Genes

Using univariate Cox regression analysis, we explored the relationship between hub gene expression levels and overall survival (OS) in LUAD patients. A prognostic signature was constructed using the least absolute shrinkage and selection operator (LASSO) Cox regression analysis for 10 prognostic-related hub genes and the coefficient of each hub gene was calculated in the TCGA cohort. The optimal penalty parameter that was calculated by 10-fold cross validation was used to filter out signatures. Risk score = sum of coefficients ^∗^ TPM value of hub genes. The formula was used to calculate a risk score for each LUAD patient in the TCGA cohort and CPTAC cohort, and patients were divided into low- and high-risk groups based on the median of the risk score from TCGA cohort. The ‘‘survival’’ package^9^ was utilized to carry out Kaplan-Meier (K-M) survival analysis for the two groups. The receiver operating characteristic (ROC) curve was generated using the ‘‘survivalROC’’ package.^10^

### Exploration of Immune-Infiltrating Cells

To explore the relationship between the level of immune cells and risk score, we calculated the immune cell status of each tumor sample in the LUAD dataset from the TCGA database using seven currently accepted methods [including XCELL (Aran, 2020), TIMER (Li et al., 2020), MCPCOUNTER (Dienstmann et al., 2019), QUANTISEQ (Plattner et al., 2020), EPIC (Racle et al., 2017), CIBERSORT (Chen B. et al., 2018), and CIBERSORT-ABS (Tamminga et al., 2020)]. Wilcoxon signed-rank test was performed to analyze immune cell differences between high- and low-risk groups as calculated by these seven methods. The correlation between the level of immune cells in tumors and risk score was analyzed using Spearman’s correlation analysis; the results are shown as a lollipop chart. The ggplot2 package^11^ was used to this procedure. *p*-Value <0.05 was considered statistically significant.

### Investigation of the Relationship Between ICI-Related Genes and Risk Score

To analyze the relationship between ICI-related immunosuppressor genes and risk score, we used the ‘‘ggstatsplot’’^12^ package to visualize the above results.

### Evaluation of the Significance of Risk Score Model in Chemotherapy and Targeted Therapy

To evaluate the clinical significance of the risk score for LUAD chemotherapy and targeted therapy, we converted the TCGA gene expression matrix into a half inhibitory centration (IC_50_) data matrix of the corresponding antitumor drugs with the “pRRophetic” package (Geeleher et al., 2014), then analyzed the IC_50_ difference between the high- and low-risk groups by the Wilcoxon signed-rank test. Results were depicted by bar chart.

## Results

### Differential Expression of Genes and the circRNA-Related Network

**FIGURE 1:**
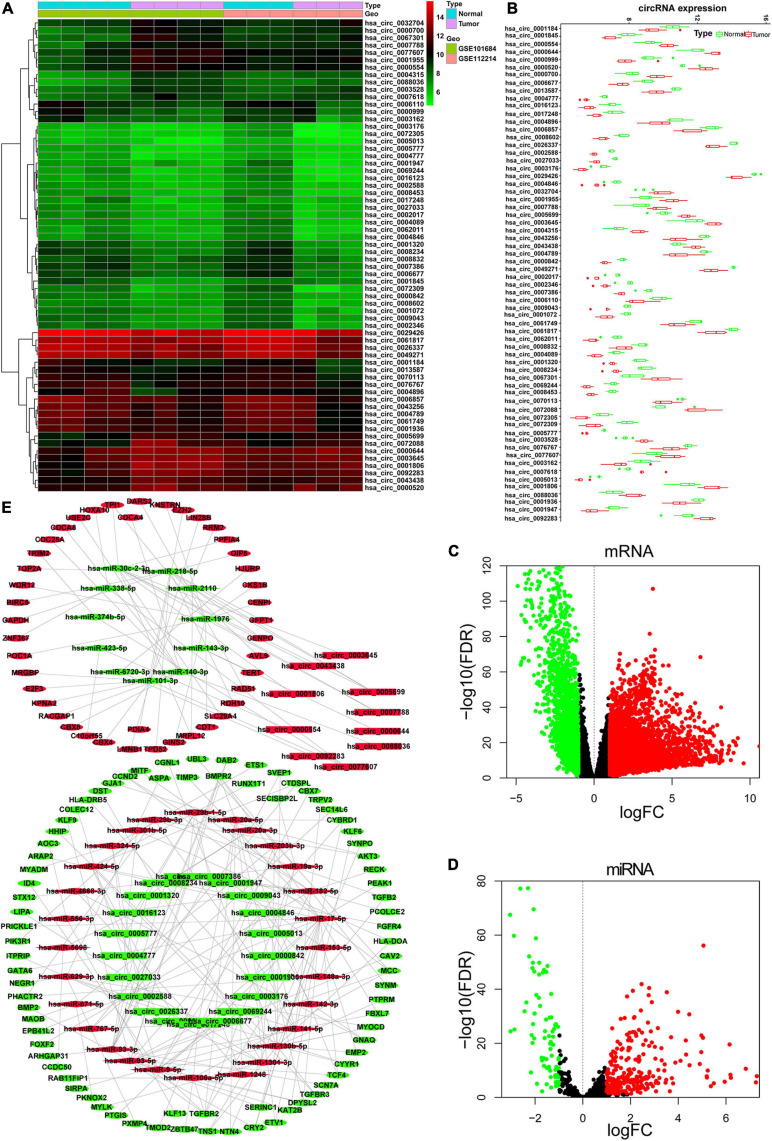
Identified DEcircRNAs, DEmiRNAs, and DEmRNAs and constructed ceRNA network. **(A)** Heat map of the 64 DEcircRNAs; the color change represents the difference in expression. **(B)** Relative expression level of 64 DEcircRNA in tumor tissue and normal tissue. Volcano plots for DEmRNAs **(C)** and DEmiRNAs **(D)**. **(E)** CeRNA network of DEcircRNAs, DEmRNAs, and DEmiRNAs. Rectangle represents circRNA; diamond represents miRNA; oval represents mRNA. Red represents upregulate and green represent downregulate. The red points and green points represent up- and downexpressed, respectively. DE, differentially expressed.

A total of 64 DEcircRNAs (18 upregulated and 46 downregulated), 362 DEmiRNAs (271 upregulated and 91 downregulated), and 5,047 DEmRNAs (3,277 upregulated and 1,770 downregulated) were identified (Figures 1A–D). To show the relationship between these DEcircRNAs, DEmiRNAs, and DEmRNAs, a circRNA-related ceRNA regulation network was constructed and visualized in Cytoscape based on the results of bioinformatics analysis (Figure 1E). Four hundred sixty-five negatively correlated (*r* < −0.2, FDR < 0.05) DEmiRNA/DEmRNA pairs (100 DEmiRNA and 297 DEmRNA) were predicted by Targetscan and miRTarBase databases. In addition, 65 circRNA/miRNA (31 circRNA and 39 miRNA) pairs predicted by circBank were constructed based on 64 DEcircRNAs and 100 DEmiRNAs with opposite expression patterns. Finally, this ceRNA network contained a total of 122 DEmRNA, 31 DEcirRNA, and 39 DEmiRNA.

### Functional Enrichment Analysis

**FIGURE 2:**
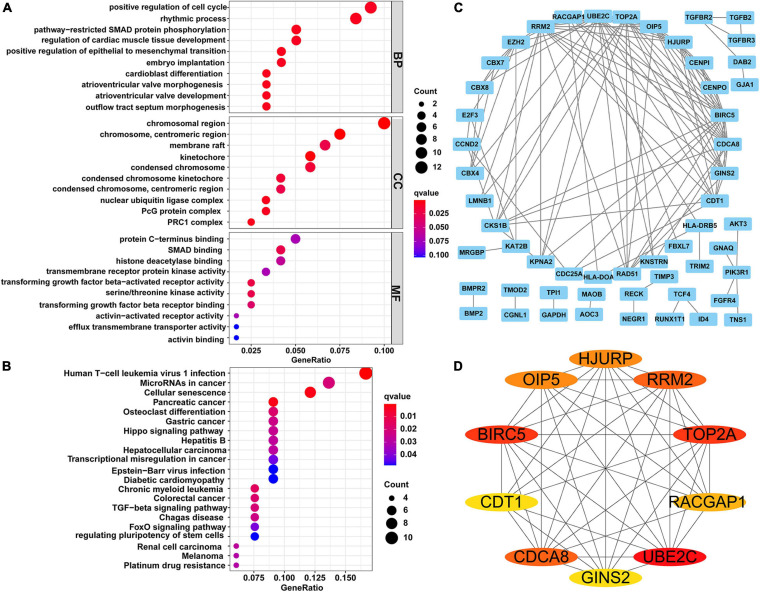
Functional enrichment analysis and constructing of PPI network. **(A)** Top 10 terms of GO function enrichment analysis. **(B)** KEGG pathway analysis. **(C)** PPI network of 122 genes, including 54 nodes and 117 edges. **(D)** The network of top 10 hub genes. Different colors represent different scores. PPI, protein-protein interaction; GO, Gene Ontology; KEGG, Kyoto Encyclopedia of Genes and Genomes.

To explore the biological functions of the identified circRNAs, we carried out GO function and KEGG signaling pathway enrichment analyses for the 122 downstream DEmRNAs regulated by circRNAs. The top 10 GO terms of BP, CC, and MF are shown in Figure 2A. The BP terms were mainly enriched in “positive regulation of cell cycle” involved in cell cycle regulation, CCs were mainly enriched in “chromosomal region” and “chromosome and centromeric region,” and MFs were mainly enriched in “protein C-terminus binding,” “SMAD binding,” and “histone deacetylase binding.” Finally, in the KEGG signaling pathway, “MicroRNAs in cancer” was the common signaling pathways for these genes. The all KEGG pathway enrichment results are shown in Figure 2B.

### Construction of the PPI Network

Using the STRING online tool, we established a PPI network for the 122 DEmRNAs to further examine their interactions (Figure 2C). This PPI network contained 54 nodes and 117 edges after removing isolated nodes. According to the degree method, the top 10 hub genes (UBE2C, BIRC5, TOP2A, RRM2, CDCA8, HJURP, OIP5, RACGAP1, GINS2, and CDT1) in the PPI network were extracted by the cytoHubba plugin (Figure 2D).

### Construction and Validation of Risk Scoring Model

**FIGURE 3:**
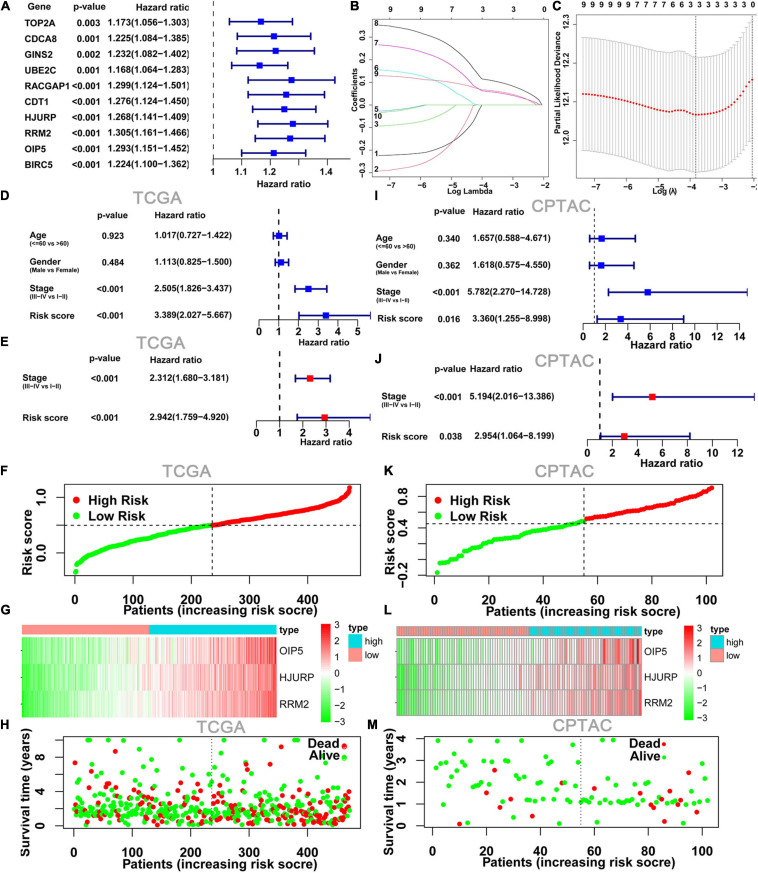
Construction and validation of risk score model. **(A)** Forest plots showing the results of the univariate Cox regression between10 hub gene and OS. **(B,C)** The minimum criteria calculated by LASSO Cox regression. Forest plot of the univariate Cox regression analyses **(D)** and multivariate Cox regression **(E)** analyses on clinical characteristics and risk score results. **(F)** Risk score plot show the risk score for each LUAD patient. **(G)** Heatmap of three hub genes from risk score model between low- and high-risk groups. **(H)** Survival status plots show survival time and status for each LUAD patient; it can be seen that the number of deaths in the high-risk group was significantly higher than that in the low-risk group. **(I–M)** The TCGA risk score model was validated with RNA sequencing data and clinical data from CPTAC program. LUAD, lung adenocarcinoma.

We next explored the relationship between 10 hub genes and OS by univariate Cox regression analysis; the 10 hub genes were identified as those with *p*-value <0.05 (Figure 3A). We then analyzed these hub genes using LASSO Cox regression analysis (Figures 3B,C). According to the minimum standard, three hub genes (HJURP, RRM2, and OIP5) were selected to build a risk score based on the risk coefficient and TPM value of genes. The risk score was calculated as follows: risk score = (0.0599 ^∗^ HJURP expression) + (0.1113 ^∗^ RRM2 expression) + (0.0652 ^∗^ OIP5 expression). K-M survival analysis also indicated that highly expressed OIP5, HJURP, and RRM2 had a lower OS in the TCGA cohort (Supplementary Figure 1). To determine whether the risk score model was an independent risk predictor for OS, we analyzed age, gender, TNM stage, and risk score by univariate and multivariate Cox regression analyses in the TCGA cohort. In the univariate Cox regression analysis model, there was a significant correlation between risk score and OS (Figure 3D). Moreover, the risk score was an independent risk predictor for OS after adjusting for other confounding factors in the multivariate Cox regression analysis model (Figure 3E).

The heatmap and survival status plots showed that the risk score was closely related to the expression levels of the three genes, and the number of deaths in the high-risk group was significantly higher than that in the low-risk group (Figures 3F–H).

To evaluate the applicability of the risk score model constructed from the TCGA dataset, the cases from the CPTAC program were also divided into low- and high-risk groups by the risk score median from the TCGA cohort. As with the TCGA results, the risk score model was an independent risk predictor in the CPTAC cohort (Figures 3I,J). And, the expression levels of these three genes and distribution of survival state in the CPTAC cohort were similar to those in the TCGA cohort in the high- and low-risk groups (Figures 3K–M).

**FIGURE 4:**
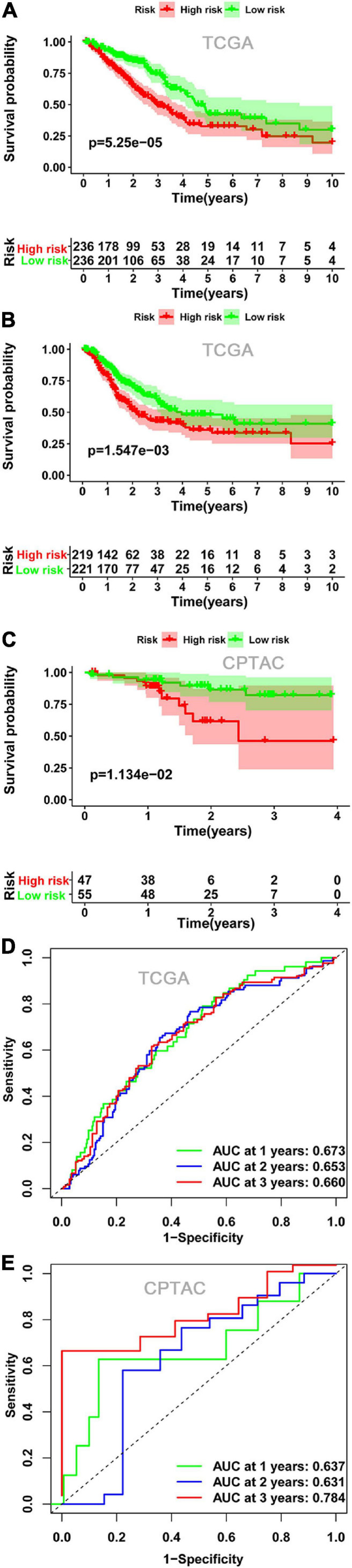
The relationship between risk score and survival. Kaplan-Meier plots of OS **(A)** and DFS **(B)** in the low- and high-risk groups in the TCGA cohort. **(C)** Kaplan-Meier plots of OS in the CPTAC cohort. ROC curve showed the prognostic value of risk score for OS in the TCGA cohort **(D)** and in the CPTAC cohort **(E)**. OS, overall survival; DFS, disease-free survival; ROC, receiver operating characteristics.

Kaplan-Meier survival analysis indicated that the OS (Figures 4A,C) and DFS (Figure 4B) in the high-risk score group were lower than those in the low-risk score group in both TCGA cohort and CPTAC cohort. Finally, we established a ROC curve of a risk score to examine its prediction power for OS. The area under the curve (AUC) of the 3-year survival data was 0.660, showing moderate accuracy and specificity in the TCGA cohort (Figure 4D). The AUC of risk score was 0.784 at 3 years in the CPTAC cohort (Figure 4E).

### Regulatory Networks for Risk Score Models

**FIGURE 5:**
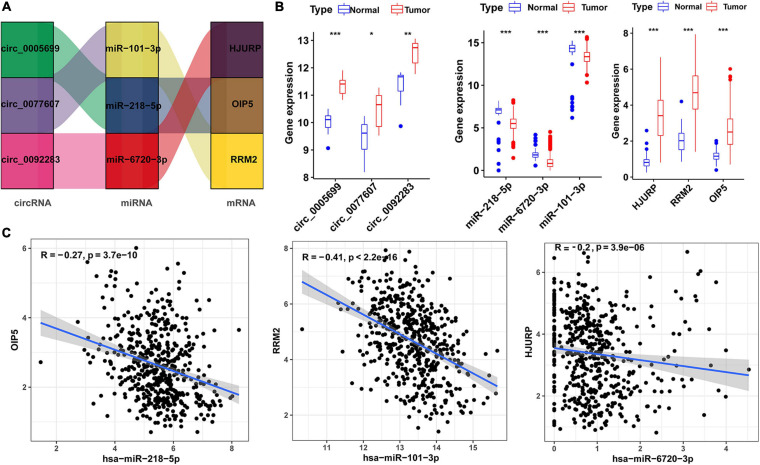
Risk score-related regulatory subnetwork. **(A)** The ceRNA subnetwork associated with risk score. **(B)** Expression level of circRNAs, miRNAs [log2(TPM)], and mRNA [log2(TPM)] in LUAD and normal samples (*p < 0.05; **p < 0.01; ***p < 0.001). **(C)** Coexpression analysis of miRNA-mRNA pairs based on TCGA data. LUAD, lung adenocarcinoma; ceRNA, competitive endogenous RNA. OS, overall survival.

To visualized the upstream genes that regulate the risk score model, we extracted ceRNA subnetwork from the total ceRNA network. This subnetwork contained three prognostic hub genes, three miRNAs (miR-101-3p, miR-218-5p, and miR-6720-3p), and three circRNAs (hsa_circ_0077607, hsa_circ_0005699, and hsa_circ_0092283) (Figure 5A). In addition, the expression level of the three circRNAs and three hub genes were upregulated in LUAD samples, while the three miRNAs were downregulated (Figure 5B). Moreover, there was a negative correlation (*r* < −0.2) between three hub genes and three miRNA expressions (Figure 5C).

### Immune Landscapes Affected by Risk Score Model

**FIGURE 6:**
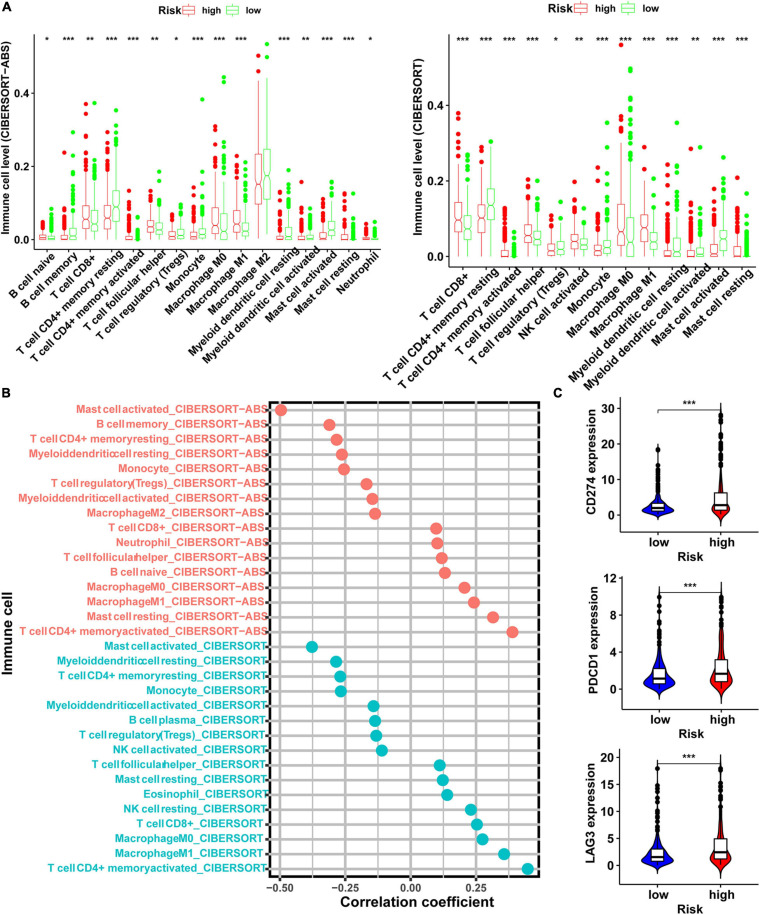
Exploration of the relationship between risk score and level of immune cells and immunosuppressive genes. **(A)** The level of immune cells in tumor evaluated by CIBERSORT-ABS and CIBERSORT software in the high- and low-risk groups. **(B)** Spearman correlation analysis of risk score and level of immune cells evaluated in CIBERSORT-ABS and CIBERSORT software. **(C)** The expression level of immunosuppressive genes in the high- and low-risk groups (*p < 0.05; **p < 0.01; ***p < 0.001).

To evaluate the associations between risk score and responses of LUAD patient to immunotherapy, we analyzed whether the risk score was associated with the types of immune cells present and ICI-related genes. Our results showed that the risk score was negatively correlated with mast cell activated, T cell CD4 + memory resting, myeloid dendritic cell resting, monocyte, T-cell regulatory (Tregs), myeloid dendritic cell activated, and NK cell activated, macrophage M2, whereas they were positively correlated with T cell CD4 + memory activated, mast cell resting, macrophage M0, macrophage M1, T cell follicular helper, and CD8 + T cells (Figure 6A and Supplementary Figure 2A). To clarify the correlation between risk score and the immune cells, we performed Spearman’s correlation analysis, and the results were shown as a lollipop chart (Figure 6B and Supplementary Figure 2B). In the same time, the result also revealed that the expression levels of genes related to ICI-related genes, such as CD274 (PD-L1), PDCD1 (PD-1), LAG3, CTLA4, and HAVCR2 were higher in the high-risk group than low-risk group (Figure 6C), but CTLA4 and HAVCR2 were not statistically different (Supplementary Figure 2C).

### Analysis of the Relationship Between the Effectiveness of Chemotherapy and Risk Score Model

**FIGURE 7:**
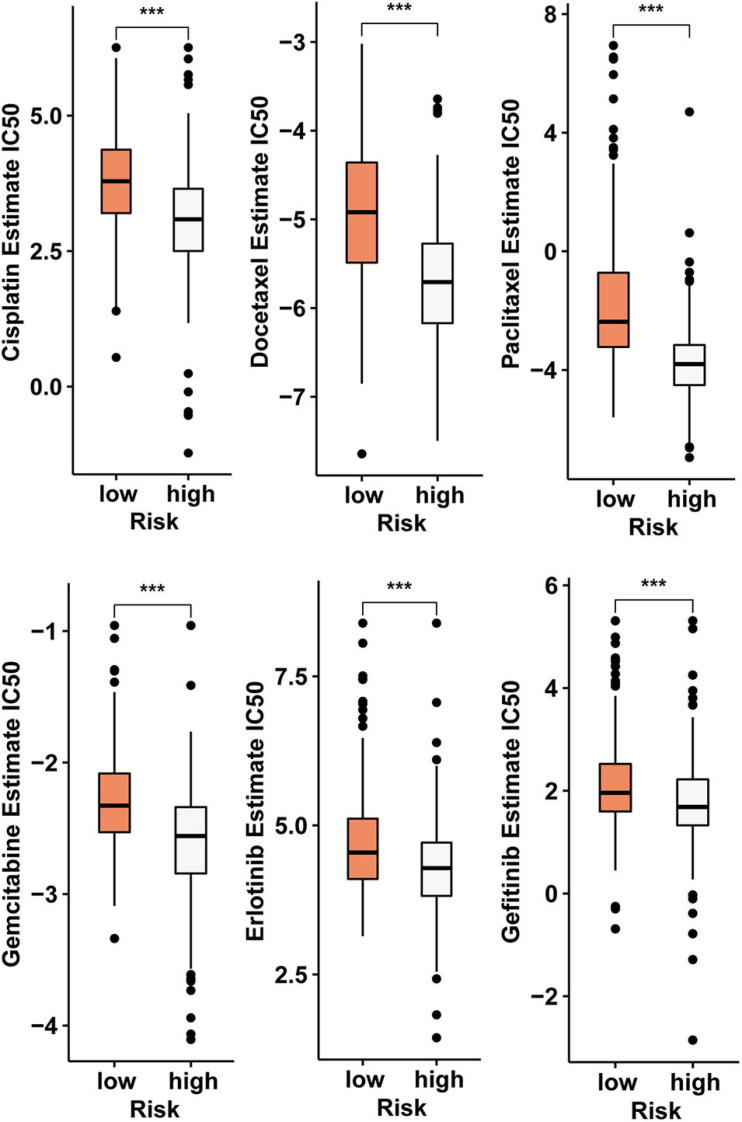
The risk score model serves as a potential predictor of sensitivity to chemotherapy and targeted therapy, as high-risk scores are associated with lower IC_50_ of chemotherapy and targeted therapy drugs such as cisplatin, docetaxel, gemcitabine, paclitaxel, erlotinib, and gefitinib (***p < 0.001).

In addition to immunotherapy, we analyzed the relationship between risk score model and the effectiveness of chemotherapy and targeted therapy in the LUAD cohort. Our result revealed that LUAD patients in the high-risk score group were more sensitive to chemotherapies such as cisplatin, docetaxel, gemcitabine, and paclitaxel and targeted drugs such as erlotinib and gefitinib. This suggests that the risk score model is a potential predictor of sensitivity to chemotherapy and targeted therapy (Figure 7).

## Discussion

Numerous studies have shown that the circRNA-related ceRNA mechanism plays a critical role in tumor function. The ceRNA mechanism hypothesis states that some non-coding RNAs, such as circRNAs and lncRNAs share miRNA response elements on mRNAs, and therefore compete with miRNAs to regulate the expression of mRNA indirectly, forming a complicated posttranscriptional regulatory network (Salmena et al., 2011). An increasing amount of evidence has shown that circRNAs are involved in several physiological and pathological processes of tumor development and progression (Song and Fu, 2019; Wang et al., 2019; Zhang et al., 2019a,b,c; Bai et al., 2020; Zhang N. et al., 2020; Zhang S. J. et al., 2020). CircRNAs have also been shown to be involved in resistance to immunotherapy, targeted therapy, and chemotherapy (Zhang et al., 2019b; Wen et al., 2020; Li et al., 2021). In this study, we identified abnormal gene expression in LUAD with data from the GEO database. According to the conjoint analysis of two databases, we then constructed a circRNA-related ceRNA regulatory network. Next, we constructed a risk score model using three mRNAs in this network and demonstrated that it is an independent risk factor for the prognosis of LUAD. Because circRNA is related to the resistance of adjuvant therapy drugs, we also analyzed the relationship between the risk score model and the status of tumor infiltrating immune cells and explored its application value in immunotherapy, chemotherapy, and targeted therapy.

In the prognostic circRNA/miRNA/hub gene subnetwork, three circRNAs (hsa_circ_0005699, hsa_circ_0092283, hsa_circ_0077607) acted as “sponges” to adsorb three miRNAs (hsa-miR-101-3p, hsa-miR-218-5p, and hsa-miR-6720-3p), thus indirectly regulating the expression level of three mRNAs (HJURP, OIP5, and RRM2) by sequestering these target miRNAs. A growing body of research has demonstrated that circRNA expression is dysregulated in lung cancer and may be related to lung cancer progression and prognosis. For example, compared with paracancerous tissue, the expression of circFGFR1 in lung cancer tissues was increased, and patients with higher circFGFR1 had a worse prognosis (Zhang et al., 2019b). Similarly, circTP63 and circular RNA100146 are highly expressed in NSCLC cells. Knockdowns of these cicrRNAs significantly inhibited tumor cell proliferation and invasion and promoted apoptosis. Further studies revealed that circTP63 and circular RNA100146 acted as a “sponges” for miR-873-3p and miR-361-3p/miR-615-5p, respectively, to suppress the expression of these miRNAs, increase FOXM1 and SF3 levels, and facilitate the progression of NSCLC (Cheng Z. et al., 2019). However, the exact mechanism of the circRNA-mediated ceRNA regulation network in LUAD is still unknown. The roles of the three circRNAs in our ceRNA subnetwork have not been reported yet. Therefore, the roles of these circRNAs need to be further confirmed in future experiments.

In the subnetwork, three miRNAs were identified, of which miR-101-3p and miR-218-5p had been previously shown to act as tumor suppressors in lung cancer. MiR-101-3p can target downstream genes to inhibit cell invasion, viability, and migration in lung cancers (Hou et al., 2017). The expression levels of miR-218 (miR-218-5p) are decreased in NSCLC tissues, and overexpression of this miRNA was shown to suppress the proliferation of NSCLC cells by regulating CDK6 (Shi et al., 2017). However, the function of miR-6270-3p in tumor has not been studied. Therefore, we can speculate that they may play critical roles in the progression of lung cancer.

According to results of LASSO Cox regression analysis for the 10 identified hub genes, OIP5, HJURP, and RRM2 were selected to establish a ceRNA subnetwork. LUAD patients with high-risk scores tend to have shorter OS and DFS. In addition, the risk score was an independent risk predictor for OS after correction for age, gender, and TNM stage. Previous work demonstrated that the expression level of OIP5 was elevated in NLCSC and esophageal cancer tissues, and silencing of OIP5 could suppress tumor cell growth (Koinuma et al., 2012). Moreover, the expression level of OIP5 was closely related to the prognosis of NLCSC and esophageal cancer and was an independent prognostic factor for LUAD (Koinuma et al., 2012). In addition to lung cancer, the expression level of OIP5 is also increased in nasopharyngeal carcinoma, and its knockout inhibits ability of the proliferation, migration, and invasion of tumor cells (Zheng et al., 2019). HJURP expression is increased in NSCLC tissues. HJURP knockdown suppressed the migration and invasion of NSCLC cells *via* inhibition of the activation of Wnt/β-catenin signaling (Wei et al., 2019). Similarly, ectopic expression of HJURP can promote proliferation, migration, and invasion of other tumor cells (Chen T. et al., 2018, 2019; Kang et al., 2020). Furthermore, high expression of HJURP is associated with poor prognosis in patients with colorectal and ovarian cancer and is an independent prognostic biomarker for those cancers (Li et al., 2018; Kang et al., 2020). Many studies have shown that RRM2 plays an important role in tumorigenesis and tumor progression. RRM2 overexpression, for example, promoted the gastric cancer invasion capacity, while its silencing inhibited the proliferation, invasion, and migration of lung cancer cells and other malignant phenotypes (Morikawa et al., 2010; Yang et al., 2019; Jiang et al., 2021). Moreover, the expression levels of RRM2 in lung cancer tissues is also closely related to the prognosis of patients and the level of tumor-infiltrating CD8 + T cells (Jiang et al., 2021).

Gene Ontology function and KEGG signaling pathway enrichment analyses for the 122 genes in ceRNA network provided insight into the pathogenic mechanism of LUAD. The most enriched BP was “positive regulation of cell cycle” that is involved in the regulation of the cell cycle. As we all know, there are more cells in the division phase in tumor cells than normal cells. KEGG signaling pathway enrichment analysis indicated that “microRNAs in cancer and cellular senescence” were significantly enriched. The relationship between miRNAs and tumors has been extensively studied (Hou et al., 2017; Shi et al., 2017; Cheng Z. et al., 2019; Zhang et al., 2019b; Li et al., 2020) and we also found a close relationship between miRNAs and LUAD. More importantly, in the ceRNA network constructed in this paper, all the 122 DEmRNAs identified were regulated by the upstream miRNAs. These results confirmed the reliability of our ceRNA network.

To construct a more effective prognostic model for LUAD patients, we carried out Cox regression analyses for age, sex, TNM stage, and risk score. The multivariate Cox regression analysis results showed that risk scores and the TNM stage were independent risk predictor factors for OS in TCGA and CPTAC cohort. Moreover, the risk score exhibited good prediction power for OS.

This model not only has good prediction power for OS but we also found that the risk score generated by this regulatory network is closely related to the state of tumor-infiltrating cells and the response to immunotherapy. It has been shown that patients with more infiltrating CD8 + T cells in tumor tissues are more sensitive to pembrolizumab (Garon et al., 2019). In this study, we evaluated the status of LUAD tumor-infiltrating immune cells in the TCGA cohort by seven common methods. Considering that these methods have their own advantages, disadvantages, and complexity, few studies have compared these algorithms. Through integration analysis, the results showed that in the high-risk score group, the level of CD8 + T-cell infiltration was higher than that in the low-risk group. This implied that the high-risk group may be more sensitive to ICIs. In addition, our analysis results also showed that the expression levels of ICIs-related immunosuppressive genes, especially PDCD1 (PD-1) and CD274 (PD-L1), were significantly higher in the high-risk group than the low-risk group. These results revealed that the risk score model can accurately predict the therapy response to ICIs.

This model can not only predicted the response of patients to immunotherapy, but also effectively predict the response of patients to chemotherapy and targeted therapy. Compared with the low-risk group, the IC_50_ values for cisplatin, docetaxel, gemcitabine, paclitaxel, erlotinib, and gefitinib in the high-risk group were lower. This means that patients in the high-risk group are more sensitive to these drugs. This risk score model is constructed using four mRNAs, and these mRNAs are indirectly regulated by five circRNAs. Therefore, these circRNAs can be considered affecting the immune landscape by indirectly regulating the risk score-constructing transcripts to affect the patient’s response to chemotherapy and targeted therapy.

There were some limitations in this study that should be considered. First, the circRNA-related ceRNA regulatory network was established based on databases and bioinformatics algorithms. These predictions need to be validated with experimental results. Second, due to the small sample size of the GEO datasets and the lack of clinical information, we were unable to assess the relationship between circRNA and survival. Lastly, as the circRNA expression data were acquired from the GEO database, we could not combine the circRNA results with miRNA and mRNA results from TCGA for circRNA/miRNA coexpression analysis of ceRNA correlation and connectivity.

In summary, the risk score calculated from the circRNA regulatory network can predict the prognosis of patients with LUAD and might be helpful in distinguishing patients who could benefit from adjuvant therapy. However, the conclusions in our study were inferred through bioinformatics analysis and needed to be confirmed by further experimental.

## Data Availability Statement

The datasets presented in this study can be found in online repositories. The names of the repository/repositories and accession number(s) can be found in the article/Supplementary Material.

## Author Contributions

HL performed statistical analyses, analyzed the data, and wrote the manuscript. JW collected the literature and analyzed the data. LZ designed the study and reviewed the manuscript. All authors approved it for publication.

## Conflict of Interest

The authors declare that the research was conducted in the absence of any commercial or financial relationships that could be construed as a potential conflict of interest.

## Publisher’s Note

All claims expressed in this article are solely those of the authors and do not necessarily represent those of their affiliated organizations, or those of the publisher, the editors and the reviewers. Any product that may be evaluated in this article, or claim that may be made by its manufacturer, is not guaranteed or endorsed by the publisher.

## Footnotes

1
https://www.ncbi.nlm.nih.gov/geo/

2
https://bioconductor.org/packages/limma/

3
https://CRAN.R-project.org/package=pheatmap

4
https://portal.gdc.cancer.gov/

5
https://proteomics.cancer.gov

6
http://www.circbank.cn/searchCirc.html

7
https://bioconductor.org/packages/clusterProfiler/

8
https://string-db.org

9
https://CRAN.R-project.org/package=survivalAnalysis

10
https://CRAN.R-project.org/package=survivalROC

11
https://CRAN.R-project.org/package=ggplot2

12
https://CRAN.R-project.org/package=ggstatsplot
