# Supplementary material for: Construction of a circRNA-Related Prognostic Risk Score Model for Predicting the Immune Landscape of Lung Adenocarcinoma
Source: Front Genet. 2021 Aug 9;12:668311. doi: 10.3389/fgene.2021.668311 (PMC8381365; doi:10.3389/fgene.2021.668311)
Supplement: Supplementary file 1 [file Data_Sheet_1.doc]

Figure(Supplement(S)1). Kaplan–Meier plots for OS of three hub-genes in TCGA cohort.

Figure(S2). The relationship between risk score and level of immune cell. (A) The level of immune cells in tumor evaluated by other five software in the high- and low-risk groups. (B) Spearman correlation analysis of risk score and level of immune cells evaluated in other five software software. (C) The expression level of immunosuppressive genes in the high- and low-risk groups.
