# Supplementary figures and images for: Construction of a circRNA-Related Prognostic Risk Score Model for Predicting the Immune Landscape of Lung Adenocarcinoma
Source: Front Genet. 2021 Aug 9;12:668311. doi: 10.3389/fgene.2021.668311 (PMC8381365; doi:10.3389/fgene.2021.668311)

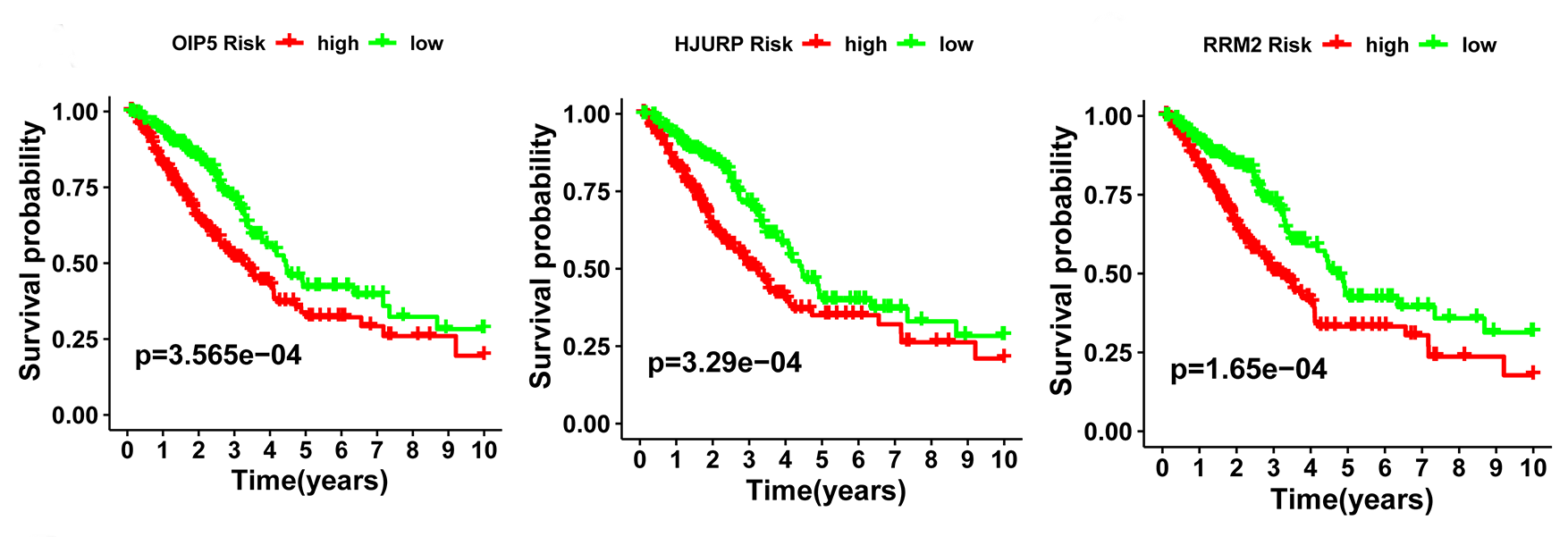

Supplement: Supplementary file 2 [file Image_1.TIF]

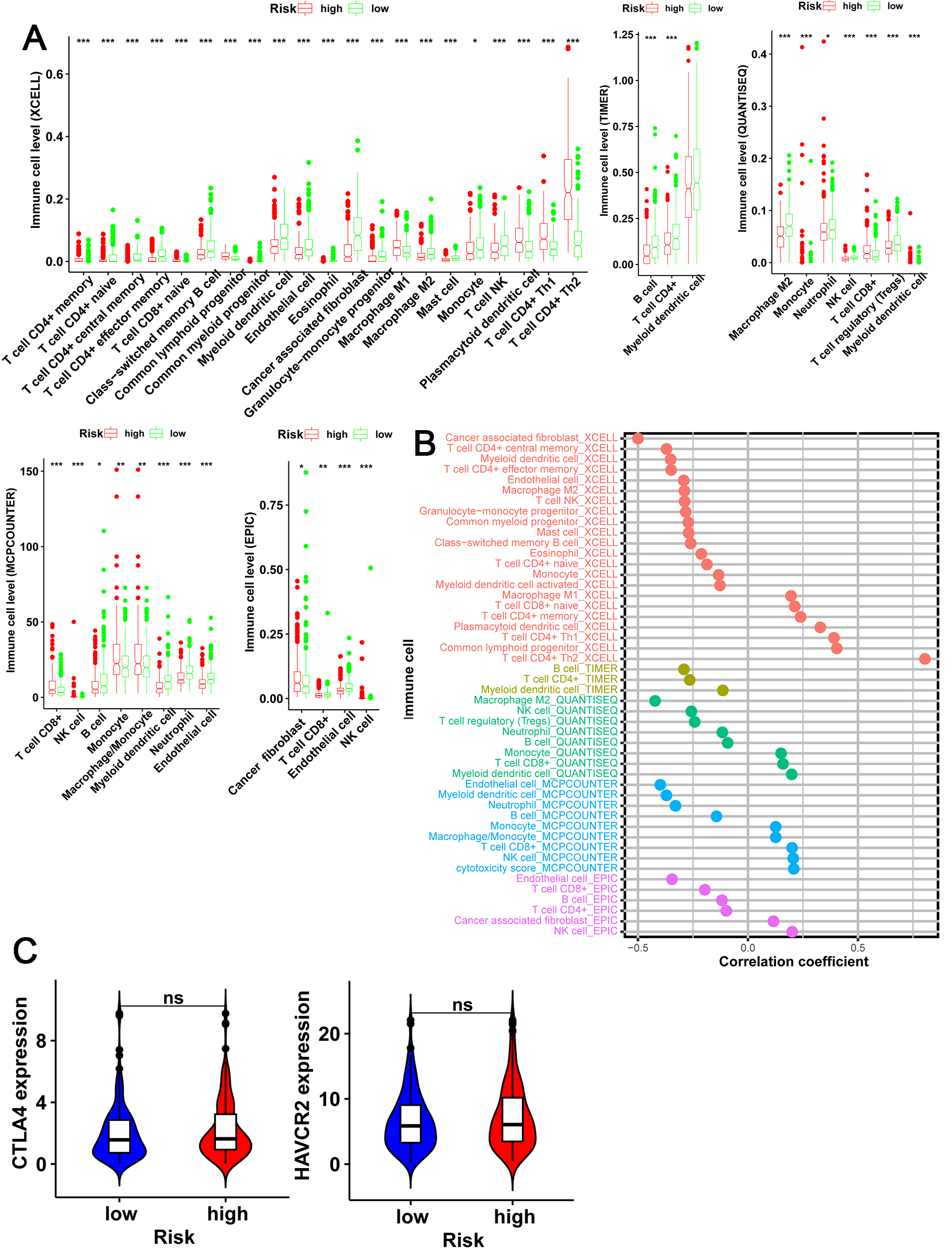

Supplement: Supplementary file 3 [file Image_2.TIF]
